# Essential Role of Rho-Associated Kinase in ABO Immune Complex-Mediated Endothelial Barrier Disruption

**DOI:** 10.3390/biomedicines9121851

**Published:** 2021-12-07

**Authors:** Hannah L. McRae, Michelle Warren Millar, Spencer A. Slavin, Neil Blumberg, Arshad Rahman, Majed A. Refaai

**Affiliations:** 1 Department of Pathology and Laboratory Medicine, Transfusion Medicine Division, University of Rochester Medical Center, Rochester, NY 14642, USA; neil_blumberg@urmc.rochester.edu; 2 Department of Pediatrics (Neonatology), Lung Biology and Disease Program, University of Rochester Medical Center, Rochester, NY 14642, USA; michelle_millar@urmc.rochester.edu (M.W.M.); spencer_slavin@urmc.rochester.edu (S.A.S.); arshad_rahman@urmc.rochester.edu (A.R.)

**Keywords:** transfusion, endothelial cells, vascular biology, ABO blood groups, immune complexes

## Abstract

ABO immune complexes (ABO-IC) formed by ABO-incompatible antigen-antibody interaction are associated with hemolysis and platelet destruction in patients transfused with ABO-nonidentical blood products. However, the effects of ABO-IC on endothelial cells (EC) are unclear. ABO-IC were formed in vitro from normal donor-derived plasma and serum. Human pulmonary artery EC (HPAEC) were cultured and treated with media, ABO-identical and –non-identical plasma, and ABO-IC. EC barrier integrity was evaluated using transendothelial electrical resistance (TEER), scanning electron microscopy (SEM), vascular endothelial (VE)-cadherin and phalloidin staining, and Rho-associated Kinase (ROCK) inhibitor treatment. TEER revealed significant/irreversible barrier disruption within 1–2 h of exposure to ABO non-identical plasma and ABO-IC; this occurred independently of EC ABO type. Treatment with ABO-IC resulted in decreased VE-cadherin staining and increased phalloidin staining in a time-dependent manner, suggesting that the resultant increased EC barrier permeability is secondary to actin stress fiber formation and loss of cell surface VE-cadherin. Inhibition of ROCK was effective in protecting against IC-induced barrier disruption even two hours after ABO-IC exposure. ABO-IC causes increased EC barrier permeability by decreasing cell surface VE-cadherin and promoting stress fiber formation, which is preventable by inhibiting ROCK activation to protect against EC contraction and gap formation.

## 1. Introduction

There is controversy among transfusion medicine specialists surrounding the definition of ABO compatibility, and there are inconsistencies in the acceptable volumes of ABO non-identical plasma transfused. Type O red blood cells (RBC), for instance, are routinely transfused to ABO non-identical recipients as they are considered compatible (i.e., universal donor) despite the small amount of plasma (up to 40 mL) in these units. However, type O plasma units (220–250 mL) would never be transfused to ABO non-identical patients as they are considered to be only compatible with type O recipients. Paradoxically, the volume of plasma within a type O platelet unit is approximately the same as a plasma unit, but this platelet unit would again be considered “universal” for an ABO non-identical recipient [1]. ABO non-identical plasma derived from platelet units has been associated with deleterious changes in platelet function in vitro and shown to cause in vivo hemolysis, at times fatal [2,3,4]. In addition, a growing body of evidence suggests that transfusing larger volumes of ABO “compatible,” but non-identical plasma is associated with an increase in complications such as sepsis, acute respiratory distress syndrome (ARDS), and mortality [5,6]. Transfusion of incompatible ABO antigen in platelet transfusions is also associated with increased bleeding and mortality in patients with intracranial bleeds [7]. Therefore, it is essential to understand the effect of even small amounts of mismatched plasma on the health of the recipient.

It has been postulated that large ABO immune complexes (IC) are formed in the transfusion recipient as a result of the interaction of donor/recipient soluble A and B antigens with anti-A and anti-B immunoglobulins. The mechanism of their formation is not well characterized, but they are thought to play a significant role in the damage associated with ABO mismatch transfusions [4,8,9]. IC formation has been shown to result in increased platelet destruction as well as platelet refractoriness, pulmonary injury, and increased morbidity and mortality [6,10,11,12,13]. Based on our current understanding of the immune response and inflammation, it is reasonable to surmise that high levels of circulating ABO IC could lead to other sequelae such as thrombocytopenia and bleeding, neutrophil activation, immune modulation, and possible injury to organs and/or tissues [1,14,15,16]; however, the involvement of IC in these phenotypes has not been well studied.

We have previously shown that ABO IC induce structural alterations in erythrocytes and platelets, both of which express ABO antigens [4]. Endothelial cells (EC) are also known to express ABO antigens, and it has been established that transfused anti-A and anti-B antibodies can cause IgG-mediated injury to EC in a mechanism similar to erythrocyte damage after exposure to ABO non-identical antibodies [17]. Therefore, we posit that understanding EC dysfunction resulting from exposure to ABO IC could deliver novel insights into the pathophysiology of transfusion-related complications such as transfusion-related acute lung injury (TRALI). Given that EC represent a key barrier between blood and tissue, maintaining vascular integrity in the case of ABO mismatch is likely important for preventing inflammation and mitigating the risk of potentially life-threatening transfusion complications.

ABO antigens have been previously reported to be among the most antigenic and immunogenic of all blood group antigens for transfusion and transplantation of solid organs (but not hematopoietic stem cells), and they are strongly expressed by the vascular endothelium [17,18,19]. Therefore, EC may also be susceptible to injury caused by ABO IC exposure as in erythrocytes and platelets. Endothelial barrier dysfunction leading to increased vascular permeability is associated with a myriad of clinical phenomena and has been implicated in the pathophysiology of several conditions including the development of atherosclerosis, cardiovascular complications in patients with chronic renal disease and/or diabetes, and acute respiratory distress syndrome (ARDS) [20,21,22]. The lung is a highly vascularized organ and is therefore susceptible to frequent interaction with blood cells and plasma proteins, particularly in the context of blood transfusion, in which the lung microcirculation is the first to encounter transfused plasma and cells. The exposure of the lung endothelium to mismatched blood products in the setting of blood transfusion may lead to vascular permeability, resulting in the accumulation of protein-rich edema and inflammatory cell infiltration. In view of this consideration, the goal of our study was to assess human pulmonary artery EC (HPAEC) barrier integrity and function in response to ABO mismatch and IC exposure.

## 2. Materials and Methods

### 2.1. Research Subject Participation

Study subject participation was sanctioned by our medical center Research Subjects Review Board (study ID 00000187), and written informed consent was provided by all participants prior to blood sample collection.

### 2.2. Blood Sample Collection

Venous blood samples were collected from healthy volunteers (n = 8, 50% female, 50% male, age 35 ± 9.8 years) of known ABO types who were free of all medications and supplements for at least seven days. Samples were collected from the antecubital vein after brief application of a light-pressured tourniquet using a 21-gauge needle. For experiments requiring platelet poor plasma (PPP) samples, eight 4.5-mL Vacutainer^®^ tubes (BD Biosciences, San Jose, CA, USA) containing a 3.2% buffered sodium citrate solution were collected from each donor. For experiments requiring serum samples, eight 4.0-mL Vacutainer^®^ SST™ tubes (BD Biosciences) were collected from each donor. Tubes were gently inverted 4–5 times immediately following collection to promote adequate mixing.

### 2.3. Sample Processing and Protein Concentration 

Sodium citrate tubes were processed 15 min after collection. PPP was obtained via centrifugation at 2200× *g* for 12 min (IEC Centra CL2, Thermo Fisher Scientific, Waltham, MA, USA). SST™ tubes were allowed to stand at room temperature for 30 min after collection and serum was then obtained via centrifugation at 2200× *g* for 12 min (IEC Centra CL2). For experiments requiring concentrated serum and plasma, samples were concentrated for 24 h using a Minicon CS15 Protein Concentrator (EMD Millipore, Darmstadt, Germany) under a sterile, ventilated hood. In parallel, stocks of group A and O normal donor plasma were retained under identical sterile conditions, without concentration, to control for storage effects on plasma proteins.

### 2.4. Measurement of Anti-A and Anti-B Titers

The isohemagglutinin titers of anti-A and anti-B were determined using samples from each donor before and after concentration using the tube technique [23,24]. Briefly, serial twofold dilutions of either plasma or serum (depending on the experiment) were prepared from undiluted to 1:2048 dilution using 0.9% normal saline. Diluted samples were incubated for 15 min at 25 °C with one drop (approximately 50–60 µL) of commercially prepared 3% RBC possessing the corresponding antigen (Biotestcell A1 & B red blood cells, Bio-Rad Medical Diagnostics GmbH, Dreieich, Germany). The samples were centrifuged for 30 s at 1000× *g* using a Hettich EBA21 centrifuge (Helmer, Noblesville, IN, USA) and evaluated for agglutination. The end-point titer was the highest dilution with 1+ agglutination. All titers were performed by a single individual to reduce variability in titer end-point determinations.

### 2.5. Immune Complex Formation

IC were formed by mixing equal volumes of either concentrated or non-concentrated group A and group O plasmas and serums, followed by incubation at 37 °C for 30 min with gentle mixing. Complex formation was confirmed by reassessing anti-ABO immunoglobulin titers after incubation using the same technique as described above. Care was taken so that anti-A and anti-B titers after IC formation were at or below the proposed “critical” level of 1:64 to control for the direct effect of residual unbound ABO antibody [25].

### 2.6. Endothelial Cell Culture

Human pulmonary artery endothelial cells (HPAEC) of known ABO types were obtained from Lonza (Walkersville, MD, USA) and cultured in gelatin-coated flasks as described [26]. Briefly, cells were grown to confluency in endothelial basal medium 2 (EBM2) containing bullet kit additives (BioWhittaker, Walkersville, MD, USA) and 10% FBS at 37 °C in a humidified atmosphere of 5% CO_2_ and 95% air. For treatment, fresh complete EBM2 media was added 1–2 h prior to challenge with serum, plasma, or immune complex. HPAEC between passages 3 and 7 were used.

### 2.7. Measurement of Endothelial Cell Permeability by Transendothelial Electrical Resistance

The endothelial barrier integrity was analyzed via transendothelial electrical resistance (TEER) across confluent HPAEC monolayers using Electrical Cell-Substrate Impedance Sensing (ECIS) (Applied Biophysics, Troy, NY, USA) as described [27,28]. Briefly, HPAEC were seeded on gelatin-coated gold microelectrodes in EBM2 containing 10% FBS. After 24 h, culture medium was replaced with EBM2 containing 1% FBS, and 2 h later, treatments of various concentrations of plasma and serum samples, IC, thrombin, and/or EBM2 were added to the wells. The TEER was then measured over a period of 8 h and normalized to baseline resistance.

### 2.8. Immunofluorescence and Confocal Microscopy

HPAEC were grown to confluency on gelatin-coated coverslips. For activation of Rho-associated kinase (ROCK) inhibitor treatment, cells were pre-treated with 10 µM Y-27632 (SCM075, Sigma-Aldrich, St. Louis, MO, USA) for 1 h. Cells were treated with plasma, serum, or IC in various concentrations (1:1 to 1:100). The treated cells were then incubated at 37 °C for varying lengths of time (from 0 to 120 min) and fixed in 4% paraformaldehyde for 15 min. The cells were then subjected to immunofluorescence staining as previously described [29]. Vascular endothelial (VE)-cadherin antibody (BD555661, BD Biosciences, San Jose, CA, USA) and goat anti-mouse Alexa Fluor 488 secondary antibody (A28175, Invitrogen, Carlsbad, CA, USA) were used to visualize adherens junctions (AJ). To localize F-actin filaments, the cells were incubated with Alexa Fluor 594-phalloidin (A12381, Invitrogen, Carlsbad, CA, USA) for 20 min at room temperature. DNA staining with Hoechst Dye was used to visualize nuclei. The coverslips were then mounted on slides using Vectashield mounting media (Vector Laboratories, Lincolnshire, IL, USA), and the images were acquired using a Zeiss Axio Imager M2m confocal microscope (Carl Zeiss AG, Oberkochen, Germany). Fiji was used to compile and edit images, and the measure tool in Fiji was used to quantitate staining intensity [30].

### 2.9. Terminal Deoxynucleotidyl Transferase dUTP Nick End Labeling (TUNEL) Assay

Confluent HPAEC monolayers grown on gelatin-coated coverslips were treated with IC diluted 1:1 with EBM-2 cell growth media for 0, 30, 60, or 120 min. Treatment media was gently removed, and cells were not washed before fixation with 4% paraformaldehyde to preserve any dead or lightly adherent cells. Cell apoptosis was assessed using the Click-iT TUNEL Alexa Fluor 594 Imaging Assay (C10246, Invitrogen, Carlsbad, CA, USA) according to manufacturer’s protocol. Coverslips were mounted on slides using Vectashield mounting media (Vector Laboratories, Lincolnshire, IL, USA), and the images were acquired using a Zeiss Axio Imager M2m confocal microscope (Carl Zeiss AG, Oberkochen, Germany). Images were compiled and edited using ImageJ. The Click-iT TUNEL assay detects the free 3′ ends of fragmented DNA, staining the nuclei of apoptotic cells. TUNEL-positive (red) nuclei and DAPI-stained nuclei were quantified and averaged.

### 2.10. Scanning Electron Microscopy

For scanning electron microscopy (SEM), HPAEC were grown on 5 mm glass coverslips, treated with media, serum, or IC, and then fixed in a 2.5% glutaraldehyde in 0.1 M sodium cacodylate buffer overnight at 4 °C. Cells were then rinsed in two changes of buffer (10 min each), post-fixed for 15 min in buffered 1.0% osmium tetroxide, dehydrated at 10 min intervals in a graded series (50 to 95%), and then in 100% ethanol. Finally, the cells were transitioned at 15-min intervals into solutions of 100% ethanol mixed with Hexamethyldisilizane (HMDS) (1:1, 1:2, 1:3), ending with four changes (15 min each) of 100% HMDS with the final change allowed to evaporate overnight in a fume hood. The dried cells on coverslips were mounted onto carbon sticky tape on top of aluminum stubs and, using a Denton vacuum evaporator, sputter coated with gold for 90 s. A Zeiss Auriga Supra Field Emission SEM (Carl Zeiss AG, Oberkochen, Germany) was used to collect digital images of the cells. Approximately 30 images were taken per treatment, and representative images are shown in the text.

### 2.11. Statistical Analysis

For TEER, the different plasma treatments (ABO-matched vs –unmatched) were compared to each other as well as to media and thrombin by their percentage change from baseline at 1 h and 8 h using a Wilcoxon test. The *p*-values for the 68 different pairwise tests were adjusted for multiple comparisons using the Benjamini–Hochberg adjustment [31], and adjusted *p*-values are denoted in the table. For confocal data and TUNEL staining, multiple groups were analyzed by one-way ANOVA, followed by Tukey post-test or two-way ANOVA. When two groups were analyzed, a Student’s *t*-test was performed. All statistical analyses were performed using GraphPad Prism 8 (GraphPad Software, San Diego, CA, USA) and data presented as mean ± SD or SE. A *p*-value of <0.05 was considered statistically significant.

## 3. Results

### 3.1. ABO Mismatched Plasma and IC Induce EC Barrier Disruption

**Figure 1:**
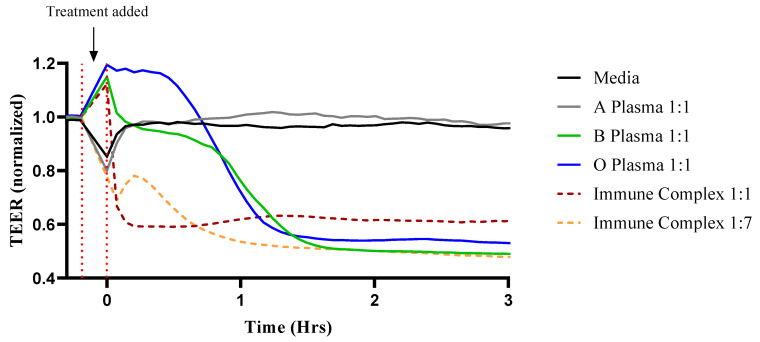
ABO mismatched plasma and IC cause endothelial barrier disruption. An illustration of type A HPAEC; EC were plated on gold electrode arrays and grown to confluency. Cells were treated with ABO identical and non-identical plasma as well as varying concentrations of IC, and transendothelial resistance (TEER) was measured over 3 h by Electric Cell-substrate Impedance Sensing System (ECIS). Resistance was normalized to the values before treatment. Dotted red bars and arrow indicate when measurement was paused and treatments were added.

We investigated the impact of mismatched ABO plasma and IC on EC barrier integrity by monitoring dynamic changes in TEER. Treating HPAEC with mismatched ABO plasma and various concentrations of IC induced EC barrier disruption within 1–2 h of exposure. In contrast, treatment with ABO identical plasma or media alone had no effect on endothelial barrier function (Table 1, Figure 1, Supplementary Materials Figures S1 and S2), suggesting that ABO incompatibility is deleterious for EC barrier integrity.

Experiments using type A, B, and O HPAEC were compiled to show that the IC effects on EC barrier are independent of the EC ABO type. The change in TEER is reported as percent change in resistance at 1 and 8 h after barrier disruption compared to baseline levels. Thrombin is a well-established proinflammatory and edemagenic agonist whose concentration is elevated in a number of diseases such as sepsis in which endothelial permeability is a major pathogenic feature. It has been widely used as a prototype agonist to model short term reversible endothelial permeability [32,33,34]. In accordance with what has been shown previously, thrombin caused a rapid decrease in TEER that recovered over time [32,35]. Interestingly, mismatched ABO plasma and IC-induced EC barrier disruption was apparent at 1 h after treatment and did not recover after 8 h. The rate of barrier disruption with IC was dose-dependent, with concentrations ranging from 1:1 to 1:100 with media (Table 2, Supplementary Materials Figure S3). These results show that ABO non-identical plasma and IC irreversibly damage the EC barrier independent of EC ABO type.

### 3.2. IC Induces EC Barrier Disruption without Causing Apoptosis

**Figure 2:**
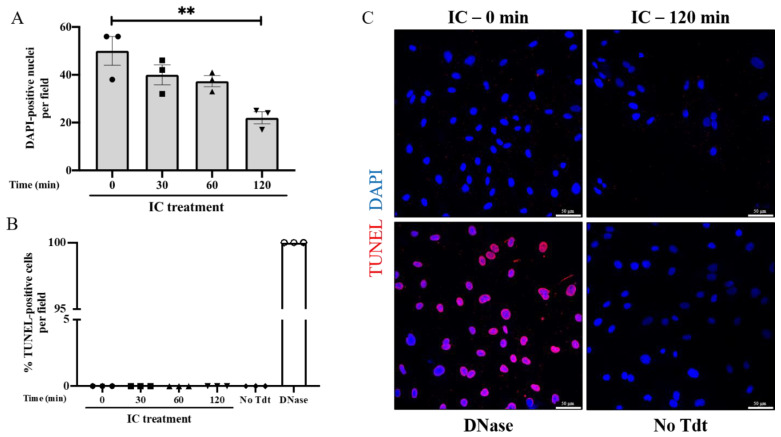
IC treatment does not cause apoptosis. Type O HPAEC treated with serum-derived IC 1:1 with media for 0, 30, 60, or 120 min. TUNEL staining was performed to mark the nuclei of apoptotic cells and DAPI was used to mark nuclei, with DNase treatment as a positive control and no Tdt as a negative control. (**A**) The number of DAPI-positive nuclei per field was averaged for each treatment to quantify the number of cells. (**B**) The percentage of TUNEL-positive cells per field for IC treatments of 0, 30, 60, and 120 min, no Tdt, and DNase controls was reported by quantifying the number of cells with TUNEL-stained nuclei normalized to the number of DAPI-stained cells per field. Note: no positive cells were seen in IC 0, 30, 60, 120, and No Tdt. (**C**) Representative images from 0 min, 120 min, DNase, and no Tdt treatments with TUNEL staining (red) and DAPI staining (blue). Data are mean + SE (n = 3 fields per condition) and were analyzed by one-way ANOVA. *p*-values as follows: ** *p* < 0.01.

The irreversible effect of ABO mismatch and IC on EC barrier integrity led us to investigate the possibility that the cells were undergoing apoptosis and thus unable to recover from injury. Type O HPAEC were treated with serum-derived IC for up to 120 min, and apoptosis was assessed by TUNEL staining. Of note, serum was used in this and other experiments as described in order to account for potential EC activation as a result of exposure to fibrinogen, von Willebrand factor, coagulation factors, and other procoagulant proteins. Consistent with the effects on barrier permeability, the number of cells per field decreased over time with IC treatment, with approximately 50% fewer ECs after 120 min of IC (Figure 2A). However, the IC-induced cell loss was not attributable to apoptosis, as no TUNEL-positive cells were observed after 0, 30, 60, or 120 min of IC treatment (Figure 2B,C). These results suggest that ABO IC does not mediate EC permeability by inducing apoptotic cell death and may instead cause detachment of EC from the monolayer [36].

### 3.3. IC Treatment Induces Cell Contraction to Cause EC Barrier Disruption

**Figure 3:**
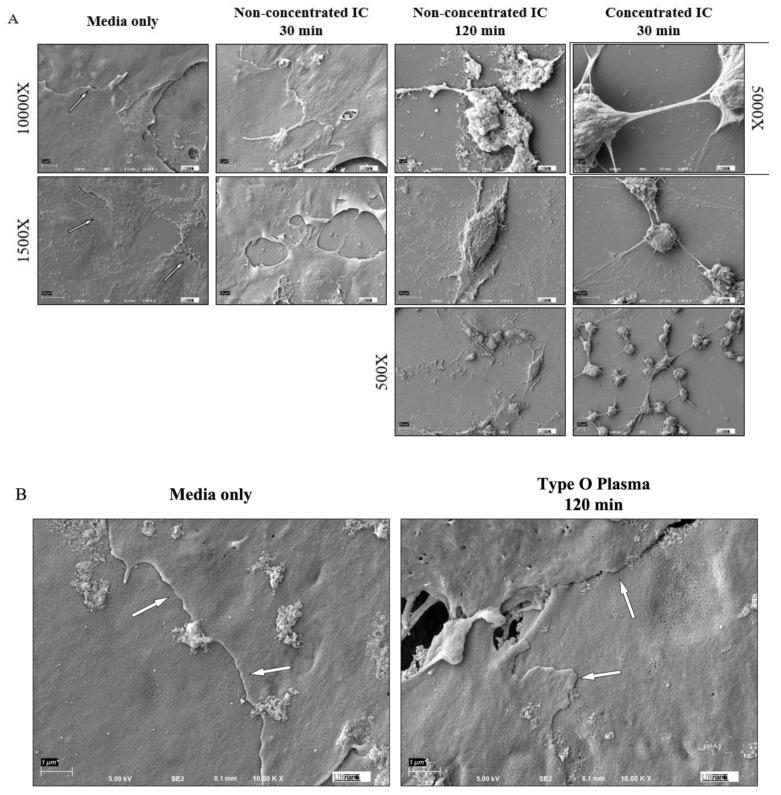
HPAEC cell–cell adhesion damaged by IC treatment. Type O HPAEC visualized by Scanning Electron Microscopy (SEM) after injury. (**A**) Cells were treated with non-concentrated or concentrated plasma-derived IC at 1:1 concentration with media for the indicated time points and imaged at different magnifications. Scale bar: 10,000×: 1 μm; 5000×: 2 μm; 1500×: 7 μm; 500×: 20 μm. (**B**) Cells were treated with type O plasma for 120 min and imaged at 10,000× as a negative control for the effects of ABO identical plasma. Arrows denote cell–cell contact sites.

To understand impact of IC on the structure of the EC monolayer, cells were visualized by scanning electron microscopy (SEM) after treatment with IC or type-matched plasma. Analysis of EC treated with non-concentrated or concentrated (i.e., having elevated anti-A titers of at least 1:64 as described earlier) plasma-derived IC revealed marked loss of cell–cell contact and rounding of cells in a concentration-dependent manner (Figure 3A). After 30 min of non-concentrated IC treatment, cell surface contact began to decrease, with gaps formed between adjacent cells, and complete dissociation occurred by 120 min. Comparatively, 30 min of concentrated IC caused almost complete loss of cell–cell contact, with only small stretches of cell membrane remaining between cells. This effect was not seen in ABO identical treatments, as type O ECs treated for 120 min with type O plasma maintained their cell–cell interactions (Figure 3B). These results support the possibility that ABO antigen antibody interaction during in vivo IC formation could disrupt endothelial barrier integrity secondary to loss of cell–cell and/or cell–matrix contact.

### 3.4. Mismatched ABO and IC Promotes VE-Cadherin Disassembly and Actin Stress Fiber Formation to Cause EC Barrier Disruption

**Figure 4:**
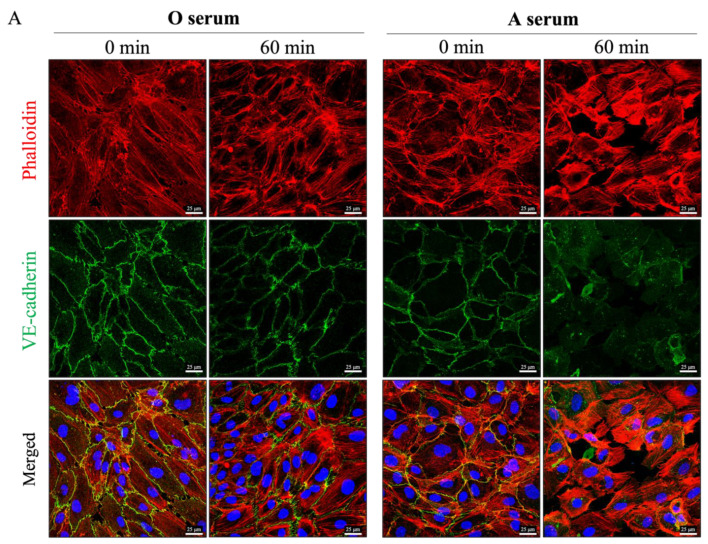

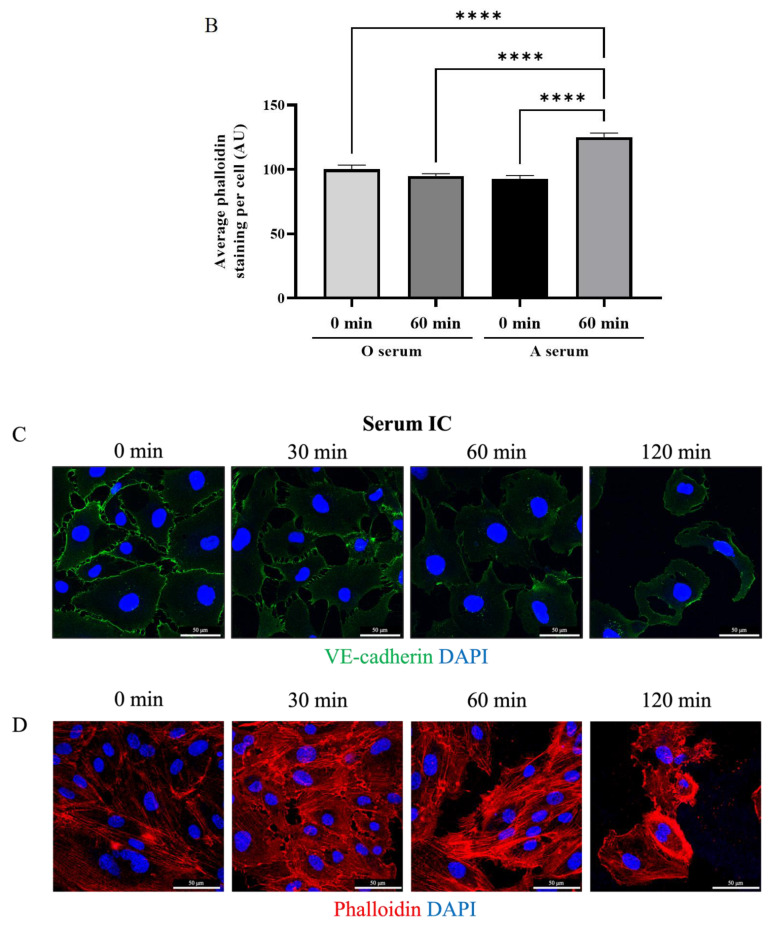

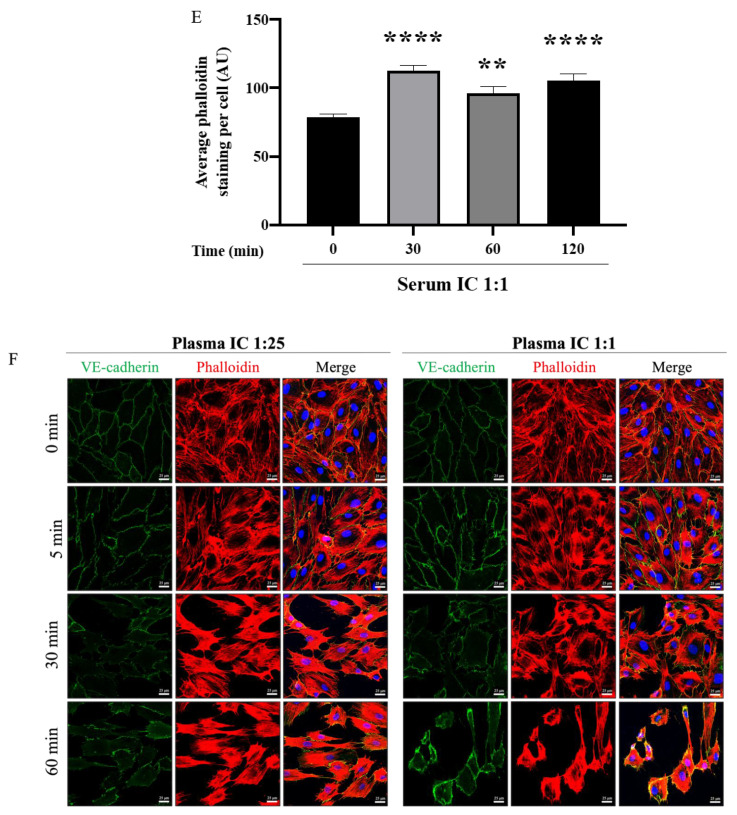

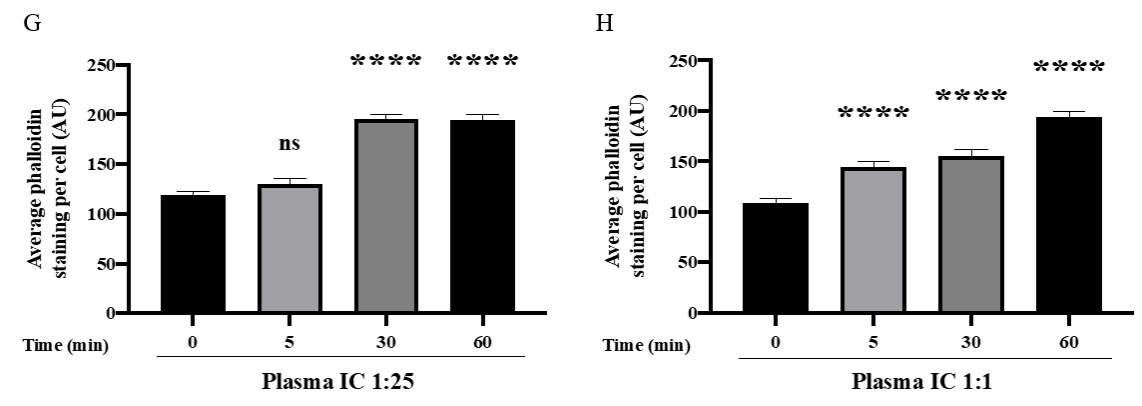
ABO mismatched serum and IC decrease cell surface VE-cadherin and induce actin stress fiber formation in HPAEC. Type O HPAEC were plated on coverslips and treated with (**A**,**B**) concentrated O serum, concentrated A serum, (**C**–**E**) serum-derived IC 1:1 with media, or (**F**–**H**) plasma-derived IC 1:25 or 1:1 with media for the indicated time points. Cells were stained with anti-VE-cadherin antibody (green) to mark adherens functions (AJs), Phalloidin-594 (red) to stain actin stress fibers, and DAPI (blue) to label nuclei. (**B**,**E**,**G**,**H**) Mean fluorescence intensity of actin stress fibers (phalloidin staining) per cell was determined. Values are reported in arbitrary units (AU) and analyzed by ANOVA. *p*-values as follows: ** *p* < 0.005; **** *p* < 0.0001; ns = not significant.

**Figure 5:**
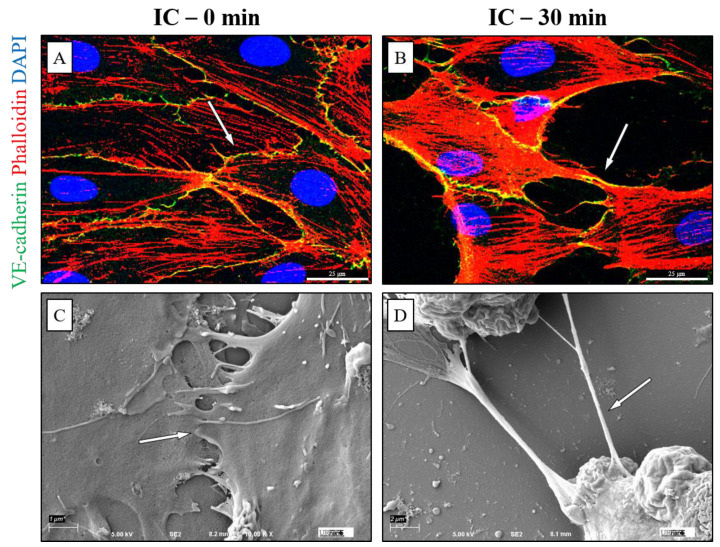
IC-induced loss of intercellular interaction visualized with confocal microscopy and SEM. Type O HPAEC were treated with (**A**,**C**) media alone or (**B**,**D**) concentrated plasma-derived IC 1:1 with media for 30 min. (**A**,**B**) Cells were stained with anti-VE-Cadherin (green) to visualize AJs, Phalloidin-594 (red) to mark actin stress fibers, and DAPI (blue) to stain nuclei and imaged at 40× and presented as merged images. Yellow color indicates regions of red and green colocalization. (**C**,**D**) Cell surface imaging was performed by SEM and viewed at 10,000× (control) or 5000× (IC). Arrows denote regions of cell surface contact between adjacent cells.

Disassembly of VE-cadherin at AJ is a key event in EC barrier dysfunction and is facilitated by cytoskeletal contraction via actin stress fiber formation. We examined cell surface VE-cadherin localization and the formation of actin stress fibers after treatment with serum- and plasma-derived IC. Both serum- and plasma derived IC led to reduced VE-cadherin staining and increased phalloidin staining in a time-dependent manner (Figure 4A–F). Interestingly, similar stretching of the cell membrane is observed in VE-cadherin- and phalloidin-stained cells as seen in SEM (Figure 5), suggesting that the stretched contacts between cells are areas where VE-cadherin has not yet been completely lost. These data suggest that ABO IC induces EC barrier permeability via actin stress fiber formation and loss of cell surface VE-cadherin.

### 3.5. ROCK Inhibition Prevents IC-Induced EC Barrier Disruption

**Figure 6:**
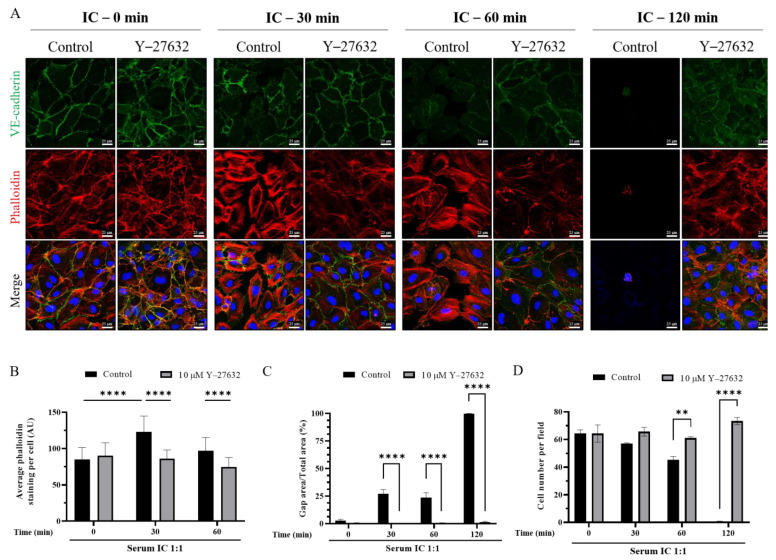
Endothelial barrier disruption by IC was prevented by treatment with ROCK inhibitor Y-27632. Type O HPAEC were pre-treated with 10 µM ROCK inhibitor (Y-27632) before injury with concentrated serum-derived IC 1:1 with media. (**A**) Cells were stained with anti-VE-Cadherin antibody (green) to mark AJs, Phalloidin-594 (red) to visualize actin stress fibers, and DAPI (blue) for nuclei. (**B**) Mean fluorescence intensity of actin stress fibers (phalloidin staining) per cell was determined. Values are reported in arbitrary units (AU) and analyzed by two-way ANOVA. The 120 min time point was excluded from statistical calculations due to low cell number in the Control group. (**C**) Gap formation between cells was measured by calculating the area without cell coverage in each field normalized to the total area of the field. (**D**) The number of cells per field was quantitated and averaged for each treatment. Data are mean + S.E. (n = 3 fields per condition) and were analyzed by two-way ANOVA. *p*-values as follows: ** *p* < 0.01; **** *p* < 0.0001.

Because ROCK is a critical determinant of actin stress fiber formation, we tested the possibility that IC destabilizes AJ via ROCK activation. Type O EC were treated with ROCK inhibitor Y-27632 for one hour before serum IC exposure and the status of cell surface VE-cadherin and stress fiber formation were assessed after 30, 60, and 120 min of treatment. As expected, control EC treated with ROCK inhibitor had reduced central stress fibers and maintained peripheral fibers [37,38,39,40]. Inhibition of ROCK was remarkably protective against IC-induced barrier disruption even up to two hours after IC exposure. EC treated with ROCK inhibitor did not form stress fibers after IC treatment and maintained VE-cadherin assembly at AJ (Figure 6A,B). Importantly, ROCK inhibition also protected against the detachment of cells caused by IC exposure (Figure 6A,D). Together these effects of ROCK inhibition prevented loss of cell–cell contact and gap formation leading to EC permeability (Figure 6C). These data suggest that IC treatment causes EC permeability by engaging ROCK, and preventing this activation is sufficient to preserve endothelial barrier function. In summary, these data demonstrate a novel effect of IC on endothelial cell permeability and identify a potential mechanism to protect against EC barrier dysfunction.

## 4. Discussion

The associations between ABO IC and other well-known adverse effects of transfusion have not been thoroughly investigated, and thus it is premature to determine the significance and prevalence of ABO IC-mediated complications, with the exception of platelet removal from the circulation, which has been demonstrated in vivo [8,9,10,11]. EC line the walls of blood vessels and serve as the barrier between circulating blood and tissue. This function is maintained by cell–cell adhesion via AJ, formed by homophilic interactions of VE-cadherin on adjacent cells [41]. Exposure of EC to noxious agents results in disruption of AJ, primarily through loss of VE-cadherin from the cell surface, which results in vascular permeability, tissue edema, and inflammatory cell infiltration into the surrounding tissue [41,42]. Loss of cell surface VE-cadherin from AJ is facilitated by contractile forces generated by actin-myosin interactions (actin stress fibers). The actin cytoskeleton associates indirectly with AJ and extracellular matrix (ECM) to support cell–cell and cell–matrix interactions and thereby stabilize the endothelial barrier [29,43]. During EC barrier disruption, however, ROCK stimulates the formation of specialized actin stress fibers. Stress fibers exert pulling forces on their associated AJ, which destabilize the EC junctions [44]. Thus, stress fiber formation and subsequent AJ destabilization lead to EC barrier permeability.

Our data suggest that the EC barrier dysfunction caused in vitro by ABO IC and mismatched ABO plasma/serum is related to the breakdown of AJ secondary to stress fiber formation and loss of cell surface VE-cadherin. We first assessed the effect of ABO IC and mismatched ABO plasma and serum on EC barrier function by ECIS. Treatment of EC with mismatched plasma and IC destabilized the endothelial barrier and increased permeability. This effect was also concentration-dependent, which is in accordance with observations that transfusing larger volumes of incompatible blood products leads to increased adverse effects [5,6]. In addition, the damage seen at a concentration of 1:100 is less than the concentration of one unit of plasma transfused to an adult patient, meaning that transfusing even individual units of ABO-mismatched plasma has the potential to cause IC-mediated harm. Importantly, exposure of EC to type-matched plasma had no significant effect on permeability, suggesting that this response is a specific result of ABO mismatch and not due to other proteins present in the plasma. Furthermore, in comparison to the damage caused by thrombin, which is known to be reversible [35], treatment with both IC and mismatched ABO plasma resulted in permanent loss of barrier integrity, and this effect does not appear to be related to apoptotic cell death. While we do not rule out necrotic cell death in this study, the ECs after IC treatment do not exhibit the swelling or plasma membrane disruption characteristic of necrosis [45], and instead display contraction and rounding. A previous study by Ge et al. observed EC detachment from the substrate in response to the fibrinogen degradation product Fragment D, which stimulated plasmin production and degradation of the extracellular matrix (ECM) [36].

The role of IC in causing EC monolayer permeability led us to investigate if the barrier-disruptive action of IC comes from its ability to disrupt cell–cell interactions. SEM was performed to visualize the EC barrier after IC treatment and revealed a concentration-dependent disruption of cell–cell interactions. Cells exposed to IC, but not type-matched plasma, lost contact with adjacent cells and became rounded. EC maintain cell–cell adhesion via interaction of cell-surface VE-cadherin at AJ between neighboring cells, and loss of VE-cadherin destabilizes these junctions, leading to increased permeability [41,43]. Staining of VE-cadherin revealed reduced cell surface localization of VE-cadherin after treatment with mismatched serum and IC. This also correlated with significant increases in stress fiber formation, which is known to facilitate the destabilization of AJ and promote EC monolayer permeability [29,44]. Importantly, EC monolayers exposed to type-matched serum maintained their cell surface VE-cadherin expression and did not form stress fibers.

Activation of actin-myosin interactions leading to contraction of EC is primarily mediated by ROCK [43,44]. To test the possibility that IC induces EC permeability via a ROCK-dependent pathway, we pre-treated cells with the ROCK inhibitor Y-27632 before IC exposure. Inhibition of ROCK by this approach prevented the permeability-inducing effects of IC exposure, protecting against stress fiber formation and disassembly of VE-cadherin, and thereby loss of cell–cell contact leading to gap formation. Strikingly, inhibition of ROCK also prevented the loss of cells. Because IC exposure did not cause apoptosis in EC, we believe the protective effect of ROCK inhibition is likely due to preservation of cell–cell and cell–matrix interactions. These data support the notion that IC causes EC barrier dysfunction via ROCK-dependent stress fiber formation, AJ destabilization, and possibly loss of cell–matrix interaction, ultimately resulting in loss of ECs from the monolayer.

The findings of this study provide novel insights into the effects of endothelial exposure to ABO IC. In addition, our data provide additional rationale for the transfusion of exclusively ABO matched blood products wherever possible, or at least avoidance of infusion of incompatible ABO antigen and antibody. This was a pilot study performed in vitro and future studies including animal models and randomized trials are needed to further investigate this phenomenon.

## Figures and Tables

**Table 1:** Percent change in TEER of HPAEC over 8 h after exposure to matched and mismatched concentrated ABO plasma as compared to media and thrombin. ABO-matched vs unmatched plasma were also compared statistically. Data shown in mean ± SD.

Treatment	N	Endothelial Cell ABO Type(s)	Percent Change at 1 h (Mean ± SD)	Percent Change at 8 h (Mean ± SD)
Media (−)	16	A, B, O	2 ± 5	3 ± 6
Thrombin (+)	6	A, B, O	20 ± 11	−17 ± 17
ABO-Identical Exposure				
A Plasma	6	A	5 ± 6	12 ± 5
B Plasma	4	B	4 ± 7	10 ± 3
O Plasma	6	O	12 ± 12	13 ± 6
ABO-Non-identical Exposure				
A Plasma	4	B	33 ± 7 *^†^	44 ± 8 *^‡^
B Plasma	6	A	38 ± 4 *^§^	51 ± 4 *^§^
O Plasma	10	A, B	36 ± 6 *^§^	49 ± 6 *^‡^

*p*-values as follows: * *p* < 0.0001 compared to media; ^†^ *p* < 0.001 compared to ABO-unmatched plasma; ^‡^ *p* < 0.005 compared to ABO-unmatched plasma; ^§^ *p* < 0.05 compared to ABO-unmatched plasma.

**Table 2:** Percent change in TEER of HPAEC over 8 h after exposure to various concentrations of IC as compared to media and thrombin. Data shown in mean ± SD.

Treatment	N	Percent Change at 1 h (Mean ± SD)	Percent Change at 8 h (Mean ± SD)
Media (−)	16	2 ± 5	3 ± 6
Thrombin (+)	6	20 ± 11	−17 ± 17
IC 1:1	26	50 ± 8 *^‡^	66 ± 9 *^‡^
IC 1:3	20	44 ± 7 *^‡^	61 ± 7 *^‡^
IC 1:7	20	42 ± 7 *^‡^	58 ± 7 *^‡^
IC 1:10	6	32 ± 11 *	69 ± 3 *^§^
IC 1:20	6	31 ± 9 *	65 ± 6 *^§^
IC 1:25	6	27 ± 8 *	64 ± 4 *^§^
IC 1:30	6	29 ± 8 *	64 ± 8 *^§^
IC 1:50	6	22 ± 93 ^†^	62 ± 5 *^§^
IC 1:100	6	11 ± 6 ^†^	51 ± 11 *^§^

*p*-values as follows: * *p* < 0.0001 compared to media; ^†^ *p* < 0.005 compared to media; ^‡^ *p* < 0.0001 compared to thrombin; ^§^ *p* < 0.005 compared to thrombin.

## Data Availability

Data sharing is not applicable to this article as no datasets were generated or analyzed during the current study.

## Supplementary Materials

The following are available online at https://www.mdpi.com/article/10.3390/biomedicines9121851/s1, Figure S1: Percent change in TEER of HPAEC over 8 h after exposure to matched and mismatched concentrated ABO plasma as compared to media and thrombin. Figure S2: ABO mismatched plasma and IC cause endothelial barrier disruption. Figure S3: Percent change in TEER of HPAEC over 8 h after exposure to various concentrations of IC as compared to media and thrombin.
