# Supplementary material for: Essential Role of Rho-Associated Kinase in ABO Immune Complex-Mediated Endothelial Barrier Disruption
Source: Biomedicines. 2021 Dec 7;9(12):1851. doi: 10.3390/biomedicines9121851 (PMC8698390; doi:10.3390/biomedicines9121851)
Supplement: Supplementary file 1 [file biomedicines-09-01851-s001.zip › biomedicines-1420377-supplementary.pdf]

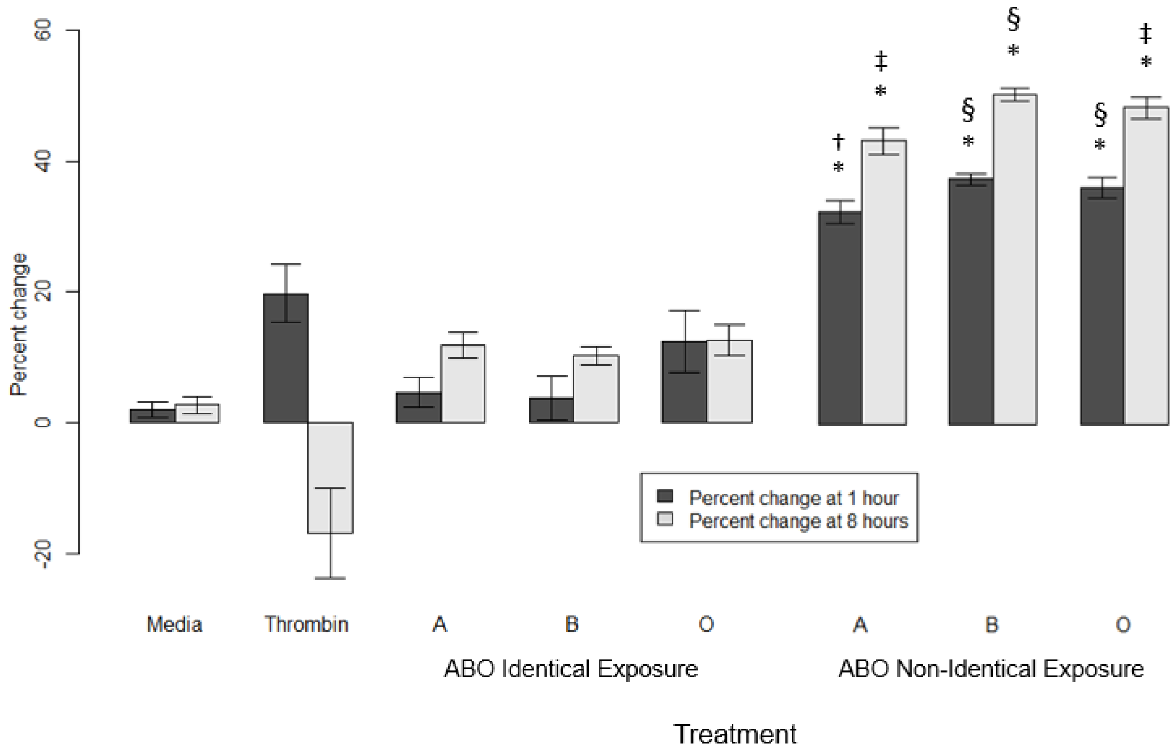

Supplemental Figure S1: Percent change in TEER of HPAEC over 8 hours after exposure to matched and mismatched concentrated ABO plasma as compared to media and thrombin. ABO-matched vs unmatched plasma were also compared statistically. P-value as follows: *p<0.0001 compared to media; †p<0.001 compared to ABO-unmatched plasma; ‡p<0.005 com-pared to ABO-unmatched plasma; §p<0.05 compared to ABO-unmatched plasma.

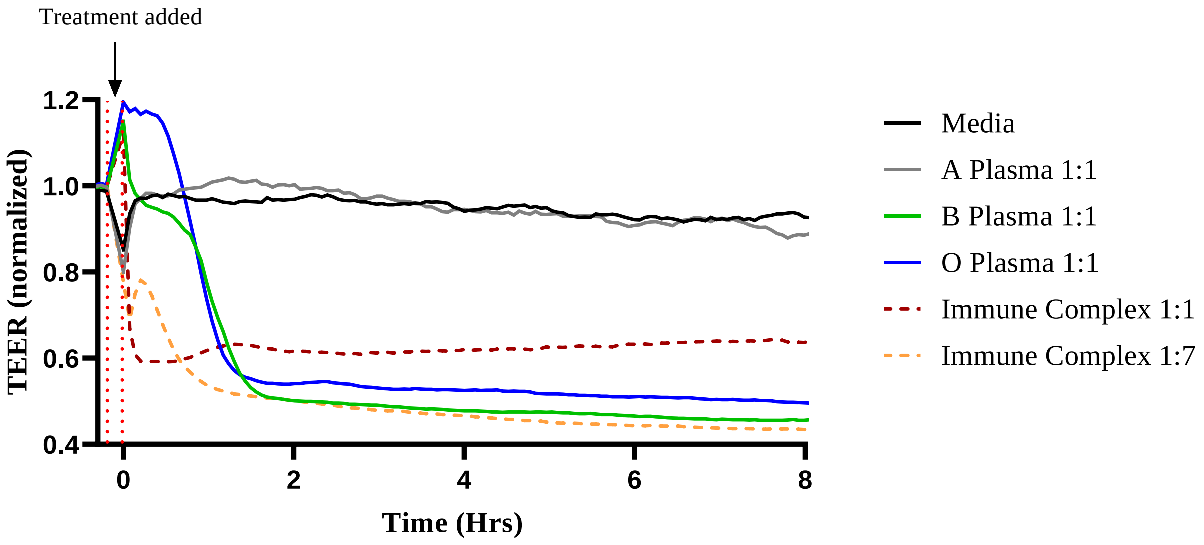

Supplemental Figure S2: ABO mismatched plasma and IC cause endothelial barrier disruption. Type A HPAEC were plated on gold electrode arrays and grown to confluency. Cells were treated with ABO identical and non-identical plasma as well as varying concentrations of IC and transendothelial resistance (TEER) was measured over 8h by Electric Cell-substrate Impedance Sensing System (ECIS). Resistance was normalized to the values before treatment. Dotted red bars and arrow indicate when measurement was paused and treatments were added.

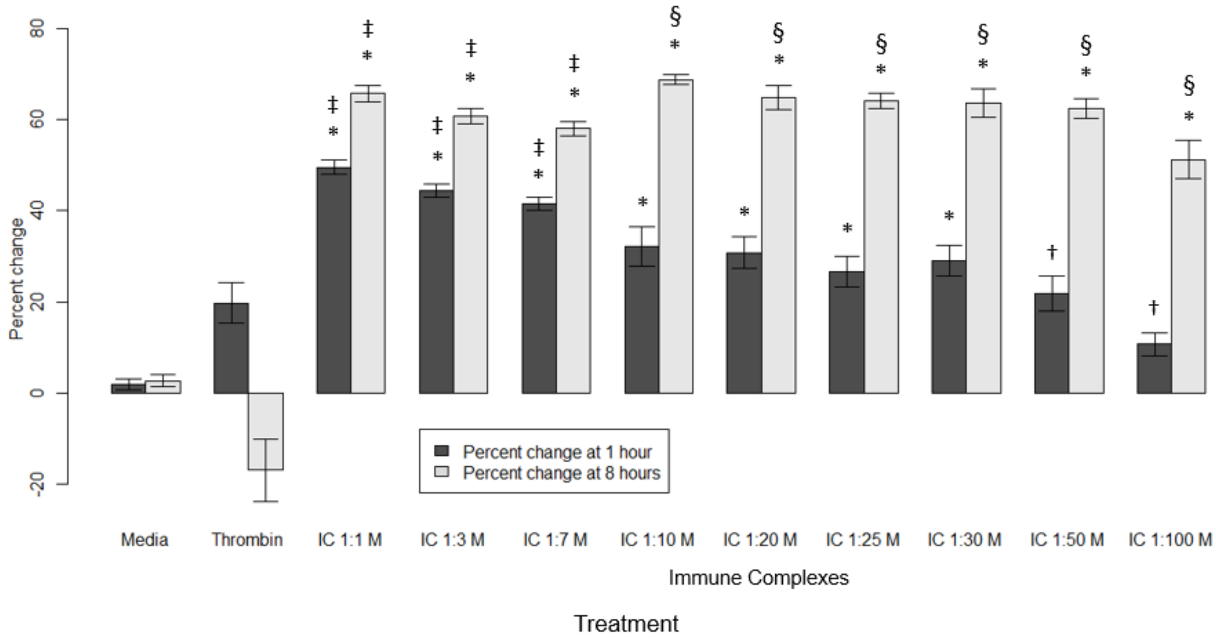

Supplemental Figure S3: Percent change in TEER of HPAEC over 8 hours after exposure to various concentrations of IC as compared to media and thrombin. P-values as follows: *$p<0.0001$ compared to media; †$p<0.005$ compared to media; ‡$p<0.0001$ compared to thrombin; §$p<0.005$ compared to thrombin.
